# Circulating levels of cell adhesion molecule L1 as a prognostic marker in gastrointestinal stromal tumor patients

**DOI:** 10.1186/1471-2407-11-189

**Published:** 2011-05-22

**Authors:** Hilke Zander, Tamina Rawnaq, Max von Wedemeyer, Michael Tachezy, Miriam Kunkel, Gerrit Wolters, Maximilian Bockhorn, Melitta Schachner, Jakob R Izbicki, Jussuf Kaifi

**Affiliations:** 1 Department of General, Visceral, and Thoracic Surgery, University Medical Center Hamburg-Eppendorf, Martinistrasse 52, 20246 Hamburg, Germany; 2 Center for Molecular Neurobiology, University Medical Center Hamburg-Eppendorf, Falkenried 94, 20251 Hamburg, Germany

**Keywords:** Gastrointestinal stromal tumor (GIST), L1, Enzyme-linked Immunosorbent assay (ELISA), progression marker

## Abstract

**Background:**

L1 cell adhesion molecule (CD171) is expressed in many malignant tumors and its expression correlates with unfavourable outcome. It thus represents a target for tumor diagnosis and therapy. An earlier study conducted by our group identified L1 expression levels in primary gastrointestinal stromal tumors (GIST) as a prognostic marker. The aim of the current study was to compare L1 serum levels of GIST patients with those of healthy controls and to determine whether levels of soluble L1 in sera could serve as a prognostic marker.

**Methods:**

Using a sensitive enzyme-linked immunosorbent assay (ELISA), soluble L1 was measured in sera of 93 GIST patients und 151 healthy controls. Soluble L1 levels were then correlated with clinicopathological data.

**Results:**

Median levels of soluble L1 were significantly higher (*p *< 0.001; Mann-Whitney U test) in sera of GIST patients compared to healthy individuals. Median soluble L1 levels were particularly elevated in patients with recurrence and relapse (*p *< 0.05; Mann Whitney U test).

**Conclusion:**

These results suggest that high soluble L1 levels predict poor prognosis and may thus be a promising tumor marker that can contribute to individualise therapy.

## Background

Gastrointestinal stromal tumors (GIST) are the most common gastrointestinal mesenchymal tumors with an incidence of 10-13 per million people a year [1]. They are mostly discovered incidentally during endoscopic or surgical procedures or are diagnosed in the evaluation of patients suffering from abdominal pain or/and upper gastrointestinal bleeding. GISTs occur most frequently in the stomach and small intestine, but rarely in the esophagus and colorectum [2,3]. GISTs are thought to arise from common precursor cells of the interstitial cells of Cajal (ICC) from the muscular plexus in the gut wall [4]. Following the consensus approach of 2002, they are classified according to tumor size and mitotic index [5]. After surgical resection the outcome of GIST is correlated with size and mitotic count and patients with recurrence have a worse prognosis [6]. Activating mutations in the tyrosine kinase *c-kit *(CD117) gene and platelet-derived growth factor receptor α gene (PDGFRα) have been reported to play important roles in the progression and diagnosis of GISTs [7,8]. First line therapy for unresectable or metastatic GISTs by molecular targeting with the tyrosine kinase inhibitor imatinib (*Glivec*^®^) has been shown in clinical trials to be efficacious and well tolerated [9].

There is to date no prognostic serum marker for GISTs. One possible candidate is the neuronal cell adhesion molecule L1 (CD171), which is expressed in primary tumors in 74% of cases [10]. L1 is a 200-220 kDa multidomain type 1 membrane glycoprotein of the immunoglobulin superfamily which plays a central role in neural development and regeneration and in synaptic plasticity in the adult nervous system [11,12]. It affects neuron-neuron adhesion and neurite outgrowth on Schwann cells as well as neurite fasciculation and myelination [13]. The extracellular domain of L1 can be regulatorily cleaved from the tumor cell surface in a process called shedding. Several L1 processing proteases could be identified such as members of a disintegrin and metallprotease ADAM10, ADAM17 [14,15] and PC5A [16]. The shed and solubilised extracellular domain is able to mediate L1 signal transduction via homo- and heterophilic interactions [17]. Among others L1 binds integrins and other members of the L1 family, such as CHL1 and NrCAM [18-20].

L1 overexpression has been detected in various tumor cell types of neural, mesothelial, and epithelial origin. Expression profile analysis in multiple human tumors has identified L1 as a molecular marker for differential diagnosis and targeted therapy [21,22]. Its presence in tumors and sera has a prognostic significance in ovarian, uterine and renal cell carcinomas and is associated with metastasis of melanomas [23-25].

The aim of this study was to evaluate whether soluble L1 could serve as diagnostic marker and prognostic factor in GIST patients by correlating L1 serum levels to long-term outcome.

## Methods

### Study design and patients

The study was approved by the Ethics Committee of the Chamber of Physicians in Hamburg, Germany. Written informed consent was obtained from all patients for using tissue and serum samples. For this study, 93 patients with GIST were chosen retrospectively. It was possible to evaluate a rather large number of patients suffering from this rare tumor because of the cooperation of a self-help organization for GIST patients called "Lebenshaus e.v.", Bad Nauheim, Germany, which helped to collect serial serum samples from patients across Germany, Austria and Switzerland. The blood samples were taken from patients at time of diagnosis and every 3 months thereafter. We selected patients on the basis of availability of specimens and did not stratify them due to rare occurrence and different treatment strategies. All data including sex, age, tumor size, mitotic count, tumor location, metastasis, relapse, and imatinib therapy were obtained from the clinical and pathological records. The clinicopathological data from externally treated patients participating in the self-help project were sent regularly by their attending physicians. As healthy controls, 151 blood bank donors were included in the study. Limitations in this study were that we had some missing items in the clinical and pathological data of our patients due to logistic difficulties in collecting data of patients from different hospitals. However, a sufficient number of GIST patients were included in the study.

### ELISA for the detection of soluble human L1

For the detection of soluble L1, 96-well flexible microtiter plates (Costar 9019) were coated with 50 μl per well of 10 μg/ml of capturing antibody (anti-L1 mAb 5G3, BD Bioscience) overnight at 4°C. Wells were blocked with 3% w/v bovine serum albumin (BSA; Fraktion V, 98% purity, Sigma Aldrich, Munich, Germany) in PBS/T (phosphate buffered saline, pH 7.3, containing 0.05% v/v Tween) for 45 min at room temperature and then incubated for 1 h with human sera diluted 1:5 in PBS at room temperature. After five washes with PBS/T, bound protein was detected with biotin-conjugated monoclonal antibody UJ127 (Dianova, Hamburg, Germany)- which does not cross-react with the close homolog of L1 (chL1) - followed by streptavidin-conjugated peroxidase using TMB (3,3', 5,5"-tetramethylbenzidine) as substrate. The color reaction was stopped by the addition of 10 mM H_2_SO_4 _and analysed at 450 nm using an ELISA reader. Human L1-Fc protein served as an internal standard for the assay.

To ensure that the immunoassay was suitable for measuring clinical serum samples, reproducibility, linearity and crossreactivity were examined. The assay showed no crossreactivity with the closed homolog of L1 CHL1, excellent linearity with serial dilutions and a low coefficient of variation (CV) in intra- and inter-assay variability.

### Sample preparation and Western blot analysis

For analysis of L1cam protein, PBS washed GIST882 cells were lysed in RIPA buffer (50 mM Tris-HCl, 150 mM NaCl, 1 mM Na_4_P_2_O_7_, 1 mM NaF, 1 mM EDTA, 2 mM Na_3_VO_4_, 1 mM PMSF, 1% NP-40 plus complete EDTA-free protease inhibitor mixture, pH 7.5) for 30 min at 4°C and then subjected to SDS-Page and Western blot analysis. Equal amount of protein of each sample was mixed with 2x Laemmli Buffer (4% SDS, 20% glycerol, 10% 2-mercaptoethanol, 0.004% bromphenol blue, 0.125 M Tris HCl, pH 6.8) and boiled at 95°C for 5 min. Protein extracts were separated by 4 - 12% SDS-PAGE (NuPage, Invitrogen, Karlsruhe, Germany) and transferred onto nitrocellulose membrane (Protran; Whatman Schleicher and Schuell Dassel, Germany). For immunoblotting, membranes were blocked with StartingBlock (TBS) Blocking Buffer (Pierce, Rockford, IL, USA) and incubated overnight at 4°C with primary 1:1000 biotinylated antibody L1-UJ127 (Dianova, Hamburg Germany). UJ127 binds to a membrane proximal Fn repeat. This allows detection of both the ADAM-cleaved 180 kDa shed fragment and the 80 kDa membrane-bound fragment presumably generated by plasmin cleavage. After washes with TBST (TBS with 0.1% Tween 20), membranes were incubated with HRP-conjugated streptavidin (Sigma Aldrich, Munich, Germany) (1:500) for 1 h at room temperature. After extensive washes, streptavidin-reactive bands were visualized using enhanced chemiluminescent substrate (ECL, Pierce) on x-ray films (Kodak Biomax-ML; Sigma-Aldrich, Munich, Germany).

### Statistical analysis

SPSS^® ^for Windows (Version 11.5.1) (SPSS^® ^Inc., Chicago, IL, USA) was used for statistical analysis. Statistical significance was evaluated by the Mann-Whitney U and Kruskal-Wallis test. Cross-table statistics were performed using Fisher's test. Recurrence-free survival was plotted using the Kaplan-Meier method and analysed by the log-rank test. Significance statements refer to p-values of two-tailed tests less than 0.05.

## Results

### Characteristics of patients and healthy controls

A total of 93 patients suffering from GIST and 151 apparently healthy controls were included in our study. The median age of GIST patients was 61 years (range 28-81 years) and 47 years (range 30-70 years) for the healthy controls. Fifty-one (55%) of the GIST patients were male and 42 (45%) female, Ninety-eight (65%) of the healthy controls were male and 53 (35%) female. Patients` characteristics and clinicopathological data are listed in Table 1.

**Table 1:** Clinicopathological characteristics of GIST patients (n = 93) and soluble L1 concentrations

Characteristics	Absolute counts (%)	Median soluble L1 concentration (25^th ^and 75^th ^percentile (ng/ml))	Mean soluble L1 concentration ± SD (ng/ml)	p-value
**Total**				
GIST	93(38)	1.4 (0.8 and 2.7)	2.1 ± 2.4	
Controls	151(62)	0.5 (0.0 and 1.0)	0.7 ± 1.4	>0.001

**GIST**				
**Sex**				
Male	51 (54.8)	1.5 (0.8 and 2.7)	2.1 ± 2.6	
Female	42 (45.2)	1.3 (0.8 and 2.4)	2 ± 2.3	0.708
**Tumor size (cm)**				
<2	8 (11)	1.4 (0.9 and 4.8)	2.9 ± 3	
2-5	21 (29)	1.7 (1 and 2.4)	2.2 ± 2.2	
5-10	21 (29)	1.2 (0.7 and 2.4)	1.9 ± 2.1	
>10	22 (31)	1 (0.4 and 2)	1.4 ± 1.4	0.305
**Mitotic count (per 50 HPF)**				
<5	27 (48)	1.1 (0.6 and 1.9)	1.7 ± 2.1	
5-10	8 (14)	1.1 (0.7 and 2)	1.3 ± 0.8	
>10	21 (38)	1.7 (1.1 and 2.6)	1.9 ± 1.2	0.258
**Tumor location**				
Stomach	52 (65)	1.4 (0.9 and 2.6)	2.0 ± 2.0	
Small intestine	23 (29)	1.5 (0.6 and 2.6)	1.8 ± 2	
Others	5 (6)	1.1 (0.5 and 3.2)	1.7 ± 1.5	0.921
**Resection margin**				
R0	52 (76)	1.4(0.8 and 2.6)	2.1 ± 0.4	
R1/2	16 (24)	1.4(1.2 and 2.6)	2.0 ± 0.4	0.529
**Recurrence**				
No	35 (45)	1.1 (0.8 and 1.7)	1.4 ± 1.2	
Yes	42 (55)	1.7 (0.9 and 3)	2.4 ± 2.3	0.049
**Imatinib**				
No	35 (46)	1.4 (0.8 and 3.0)	2.4 ± 2.7	
Yes	41 (54)	1.5 (0.7 and 2.4)	1.6 ± 1.2	0.673
**Risk NIH criteria**				
Very low	5(9)	1.2 (0.7 and 2.8)	1.7 ± 1.4	
Low	9(16)	1.1 (0.8 and 1.9)	1.4 ± 1.0	
Intermediate	9(16)	0.7 (0.3 and 2.4)	2.0 ± 3.0	
High	33(59)	1.5 (0.8 and 2.4)	1.7 ± 1.4	0.834
**Risk AFIP criteria**				
Very low	5(9)	1.2 (0.7 and 2.8)	1.7 ± 1.4	
Low	11(20)	1.4 (0.9 and 2.2)	1.8 ± 1.4	
Intermediate	13(23)	0.7 (0.2 and 2.0)	1.9 ± 2.8	
High	27(48)	1.5 (1.0 and 2.6)	1.6 ± 1.1	0.717

NIH: National Institute of Health; AFIP: Armed Forces Institute of Pathology

Not all counts add up to the full number of included patients (n = 93), due to incomplete data. P-values were calculated by the Mann-Whitney-U and Kruskal-Wallis tests

### Soluble-L1 concentrations of healthy controls and GIST patients

**Figure 1:**
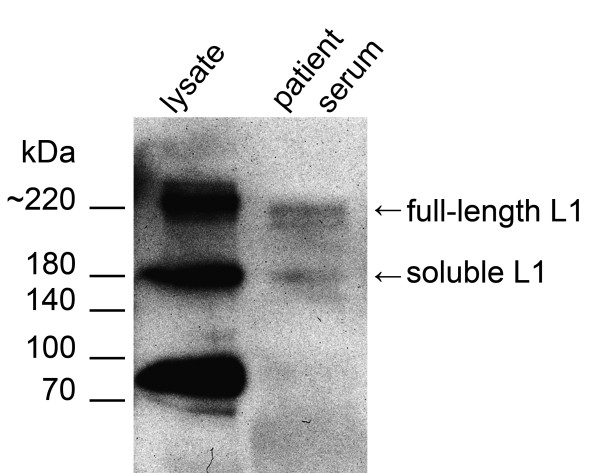
**Western blot analysis of GIST882 cell lysate compared to a patient serum using the biotinylated antibody L1-UJ127**. Three major bands corresponding to L1 are detectable in the cell lysate. The 220 kDa form represents the full length L1-Protein, while L1-180 kDa and L1-80 kDa are proteolytically cleaved. L1-180 kDa represents the shedded extracellular domain while L1-80 kDa is a c-terminal fragment integral to the membrane. In serum, soluble forms of L1 with the molecular weights of approx. 200 kDa and 180 kDa are detectable. The L1-80 kDa fragment is not present in sera.

L1 could be detected in sera of GIST patients, representatively shown in Figure 1. In sera proteolytically cleaved soluble L1 fragments as well as full-length L1 were specifically recognised by the monoclonal antibody UJ127, which was also used in the ELISA, underlining the specificity of the assay. Lysate of the cell line GIST882 [26] was used to visualise membrane bound or associated L1 compared to soluble cleavage products. In patient sera, the shed extracellular domain of 180 kDa, generated by cleavage of ADAM10/17, is detectable but not the 80 kDa form generated by PC5 or Plasmin.

**Figure 2:**
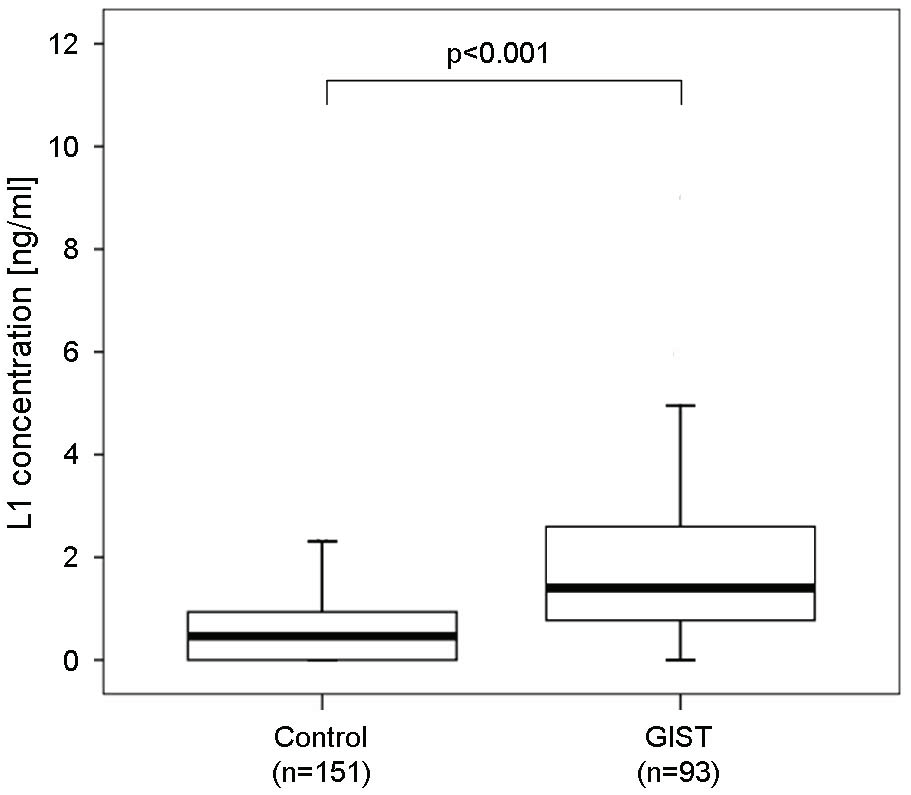
**Box-plots show medians (black lines) of soluble L1 concentrations in healthy controls (n = 151) and GIST patients (n = 93) determined by ELISA**. Statistical significance was determined by the Mann-Whitney U test (p < 0.001).

Soluble L1 (s-L1) concentrations in the sera of GIST patients were significantly higher than in healthy controls: median (25th and 75th percentiles) soluble L1 values were 1.4 (0.8 and 2.7 respectively) ng/ml in the GIST patients and 0.5 (0 and 1 respectively) ng/ml in the controls (p < 0.001; Mann-Whitney U test) (Figure 2). The mean (± SD) soluble L1 concentration was 2.1 ± 2.5 ng/ml for GIST patients and 0.7 ± 1.4 ng/ml for healthy controls. Detailed clinicopathological data are given in Table 1. In addition s-L1 levels were not elevated in other benign and malignant gastic tumors (n = 59, data not shown). There was no influence of sex, age, tumor size, mitotic count, tumor location, resection margin or imatinib treatment on soluble L1 concentrations.

### Increased soluble L1 serum concentrations correlate with recurrence and negative outcome

Table 1 shows that GIST patients with recurrence had significantly higher median soluble L1 concentrations than those without. Soluble L1 concentration (25th and 75th percentiles) was 1.7 (0.9 and 3) ng/ml in GIST patients with recurrence and 1.1 (0.8 and 1.7) ng/ml in relapse-free patients (p = 0.049; Mann-Whitney U test).

**Figure 3:**
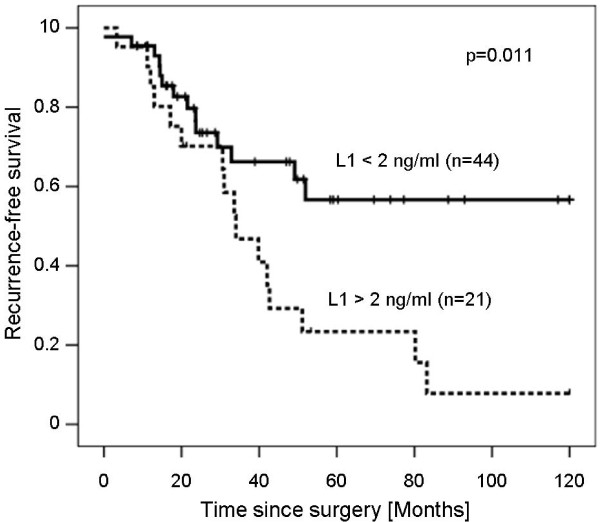
**Kaplan-Meier survival curve for recurrence-free survival in GIST patients**. Groups with soluble L1 concentrations lower or higher than 2 ng/ml were compared. P-values were calculated with the log-rank test.

The median follow-up time in the study was 37 months (range 0-273 months). For additional statistical analysis, patients with high (>2 ng/ml) and low (<2 ng/ml) soluble L1 concentrations were compared by survival curves plotted by the Kaplan-Meier method for recurrence-free survival (Figure 3). GIST patients with high soluble L1 levels showed a significantly worse outcome (38 months [95%-CI: 27; 50] of recurrence-free survival compared to 73 months [95%-CI: 56; 90] in patients with low soluble L1 levels (p = 0.016, log-rank test). Accordingly, the five-year recurrence-free survival rate among patients with low soluble L1 levels was 52%, while it was only 19% in patients with high soluble L1 levels. Absolute recurrence counts were significantly higher in patients with high soluble L1-levels (p = 0.025, Fischer's test, data not shown). We refrained from analysing overall survival because of low numbers (n = 4) of deaths in the patient group in this study.

Neither mitotic count nor tumor size had an influence on recurrence-free survival plotted by Kaplan-Meier analyses (p = 0.231 and p = 0.199, log rank test; data not shown). Also mitotic count and tumor size could not be significantly correlated with recurrence (p = 0.075 and p = 0.725, Fisher's test).

## Discussion

Most primary cancers develop loss of expression of adhesion molecules. This allows for a critical step in metastasis to occur: detachment of the invading cell from its neighbors [27,28]. Some adhesion molecules, such as L1, however show elevated expression in some tumor entities. There are several potential interpretations for this phenomenon. Increased homophilic intercellular adhesion may favor the metastatic process because cell aggregates, rather than single cells breaking away from the primary tumor, have a greater chance of surviving in the circulation and of lodging in other organs [29]. Furthermore it is known that cell adhesion is necessary for the metastatic spread of cancer cells to new organs [27]. It was shown that L1 expressed on tumor endothelium could favor metastasis and angiogenesis [30]. The development of new blood vessels is a key step of tumor progression that might be promoted by the interaction of proteolytically cleaved soluble forms of L1 with integrins [31]. The shed L1 can promote L1 dependent homophilic stimulation in an autocrine fashion [32]. The proteolytic events not only take place on the cell surface but also on membrane vesicles such as exosomes and apoptotic membrane vesicles [33]. This can explain the detection of full length and shed L1 in sera.

In a previous study we could show that L1 expression was immunohistochemically detectable in 74% of GISTs, but not in differential diagnostic important tumors such as smooth muscle tumor, peripheral nerve sheath tumor or desmoid-type fibromatosis. Although c-kit expression is the etablished standard in the identification of GIST, additional prognostic markers are beneficial. To date, most studies regarding L1 expression have been performed at the mRNA level or by immunohistochemistry. To evaluate the role of L1 as a peripheral marker, we here examined L1 levels in serum of GIST patients and healthy controls.

Using a specifically developed robust and highly sensitive immunoassay we were able to demonstrate that soluble L1 concentrations were significantly elevated in the blood of GIST patients compared to a healthy control group and other gastric tumors. Furthermore, we observed a significant association between increased soluble L1 levels and recurrence, which predicts worse outcome in GIST. Patients with high soluble L1 concentrations had a poorer recurrence-free survival than patients with lower levels. These observations are in agreement with our previous immunohistochemical study, where a trend towards worse outcome of patients with L1-positive tumors was reported [10,34]. Elevated soluble L1 as a negative prognostic marker has already been shown for some types of ovarian cancers [25]. In melanoma and ovarian carcinoma the cleaved L1CAM ectodomain can play a role in tumor progression, conferring cellular properties that are important in advanced stages of cancer progression including enhanced motility and invasivenes [33,35]. Our study suggests that also in GIST, high L1 serum levels are leading to high cell dispersal of the tumor which augments the risk of recurrence.

Soluble L1 concentration could thus become a useful marker for tumor progression and metastasis, which predicts prognosis in GIST.

Our results also suggest that L1 may be an important therapeutic target in the treatment of GIST. The efficiency of L1 monoclonal antibody treatment in suppressing tumor growth has been described in nude mice, where intraperitoneal injection of an L1 antibody was found to inhibit intraperitoneal tumor proliferation and dissemination of malignant tumor cells [36]. In this context it might be relevant that soluble L1 levels depend on the presence of proteinases. Matrix metalloproteinases (MMPs) and a disintegrin and metalloproteinases (ADAMs) are involved in many aspects of tumor biology. ADAM17, which has been shown to mediate L1 cleavage [15] has also been identified as a major sheddase in GIST [37].

## Concusions

We provide evidence that analysis of serum concentrations of soluble L1, comprising the extracellular domain of the L1 molecule, represents a novel and useful approach to assess the malignancy and metastatic potential of GISTs. The availability of a reliable immunoassay, such as the one developed for this study, for measuring serum L1 concentrations will facilitate further studies to establish the clinical applicability of this marker in GISTs and other tumors. These studies should notably investigate L1 levels in other serum samples such as obtained pre- and post-surgery and serially during the course of therapy. Beyond clinical applicability, future work must address mechanistic questions about the functional role of L1 in the tumorigenesis of GISTs and other L1 expressing cancers.

## Competing interests

The authors declare that they have no competing interests.

## Authors' contributions

HZ developed the immunoassay and drafted the manuscript, TR participated in the design of the study and performed the statistical analysis, MW carried out the immunoassays, MT and MK participated in the statistical analysis and collected the clinicopathological data, GW performed the Western Blot, MB helped to recalculate the statistics for the revision, MS and JRI participated in its design and coordination and helped to draft the manuscript. JTK conceived of the study. All authors read and approved the final manuscript.

## Pre-publication history

The pre-publication history for this paper can be accessed here:

http://www.biomedcentral.com/1471-2407/11/189/prepub
